# Tyrosine Kinase Inhibition Regulates Early Systemic Immune Changes and Modulates the Neuroimmune Response in α-Synucleinopathy

**DOI:** 10.4172/2155-9899.1000259

**Published:** 2014-09-30

**Authors:** Michaeline L. Hebron, Irina Lonskaya, Paul Olopade, Sandra T. Selby, Fernando Pagan, Charbel E-H Moussa

**Affiliations:** 1 Department of Neuroscience, Laboratory for Dementia and Parkinsonism, Georgetown University Medical Center, Washington D.C., 20007, USA; 2 Department of Oncology, Lombardi Cancer Center, Georgetown University Medical Center, Washington D.C., 20007, USA; 3 Neurorestoration Group, Movement Disorders Program, National Parkinson Foundation Center of Excellence, Georgetown University Hospital, Washington D.C., 20007, USA; 4 Deparment of Neurology, Georgetown University Hospital, Washington D.C., 20007, USA

**Keywords:** Microglia, Tau, α-Synuclein, Nilotinib, Bosutinib, Inflammation, Abl

## Abstract

**Objectives:**

Neuro-inflammation is common in α-Synucleinopathies and Tauopathies; and evidence suggests a link between the tyrosine kinase Abl and neurodegeneration. Abl upregulates α-Synuclein and promotes Tau hyper-phosphorylation (p-Tau), while Abl inhibitors facilitate autophagic clearance.

**Methods:**

A model of α-Synucleinopathy harboring human mutant A53T α-Synuclein and exhibits concomitant increase in murine p-Tau was used to determine the immunological response to Abl inhibition.

**Results:**

Age-dependent alterations of brain immunity, including loss of IL-10 and decreased levels of IL-2 and IL-3 were observed in old A53T mice. Brain CCL2 and CCL5 were decreased, but CX3CL1 remained constantly elevated. Young A53T mice exhibited differential systemic and central immune profiles in parallel with increased blood markers of adaptive immunity, suggesting an early systemic immune response. Tyrosine kinase inhibitors (TKIs), including nilotinib and bosutinib reduced brain and peripheral α-Synuclein and p-Tau and modulated blood immunological responses. TKIs did not affect brain IL-10, but they changed the levels of all measured blood immune markers, except CX3CL1. TKIs altered microglia morphology and reduced the number of astrocyte and dendritic cells, suggesting beneficial regulation of microglia.

**Conclusions:**

These data indicate that tyrosine kinase inhibition affects neuro-inflammation via early changes of the peripheral immune profile, leading to modulation of the neuro-immune response to α-Synuclein and p-Tau.

## Background

Inflammation is reported in several neurodegenerative diseases, including Parkinson (PD), Alzheimer (AD) and the Tauopathies [1]. The inflammatory response is thought to generally localize to areas of central nervous system (CNS) injury via communication between immune cells and stressed neurons. It was initially thought that α-Synuclein-related pathology was confined to within neurons, but recent work suggests that microglia are activated following the release of α-Synuclein into the extracellular space [2]. Aggregated forms of α-Synuclein induce microglia activation [3,4], suggesting that this may be one of the mechanisms of neurodegeneration [5,6]. Activated microglia are present in postmortem brains of patients with primary Tauopathies, including fronto-temporal dementia with parkinsonism linked to chromosome-17 (FTDP), progressive supranuclear palsy (PSP) and corticobasal degeneration (CBD) [7–9]. We demonstrated microglia activity and p-Tau accumulation in α-Synuclein gene transfer models [10] and conversely, α-Synuclein phosphorylation and accumulation together with p-Tau in lentiviral Tau models [10,11]. Cell culture models also demonstrate that pro-inflammatory cytokines can induce p-Tau [12–14]; and the endotoxin lipopolysaccharide (LPS) promotes inflammation and p-Tau accumulation [15], while suppression of microglia activity prolongs survival in Tau mutant P301L transgenic mice [16]. These findings suggest that microglia activity is associated with p-Tau and α-Synuclein through an underlying mechanism moderating communication between microglia and neurons.

The non-receptor tyrosine kinase Abelson (Abl) has been linked to inflammation in neurodegenerative diseases and animal models of neurodegeneration [17–19]. We demonstrated that Abl activation increases α-Synuclein levels in mutant A53T mice and α-Synuclein gene transfer models [20,21]. Recent findings in rat models showed that Abl activation is also associated with α-Synuclein phosphorylation [22], which may be linked to protein aggregation [10]. In AD, Abl is also associated with neurofibrillary tangles (NFTs) [23–26], and it is activated in the hippocampus and entorhinal cortex in post-mortem brains [23,24]. Src tyrosine kinase is also recognized in AD via interaction with Tau [27–29]. We demonstrated that tyrosine kinase inhibitors (TKIs), including nilotinib and bosutinib penetrate the brain and inhibit Abl, resulting in a decrease of α-Synuclein [20–22,30] and p-Tau levels [20,21,30–33]. Nilotinib (AMN107) is a second generation selective Bcr-Abl inhibitor, which is clinically effective in adult chronic myeloid leukemia (CML) [34]. Because Src and Abl are structurally homologous, the dual Src/Abl TKI bosutinib (SKI-606) can also inhibit Abl [35].

Microglia activation has been extensively studied in the pathogenesis of neurodegenerative disorders; and the role of adaptive and innate CNS immunity is a growing area of interest. However, determining the temporal interaction between systemic and CNS immunity in response to protein accumulation is critical to understanding the beneficial or detrimental role of microglia activity at different stages of disease. These studies investigated the temporal changes of peripheral and CNS innate and adaptive immune response in mutant A53T α-Synuclein mice that exhibit murine p-Tau accumulation [21,36] with and without TKI-induced reduction of α-Synuclein and p-Tau. We followed the same treatment paradigm that we previously published using 10 mg/kg nilotinib or 5 mg/kg bosutinib every other day for 6 weeks [20–22,30]. We found that peripheral systemic inflammatory markers, reflecting the interplay between innate and adaptive immunity, change in parallel with CNS immunity; and these changes are reversed by nilotinib and bosutinib. The current studies suggest communication between peripheral and CNS immunity to modulate the inflammatory brain response to α-Synuclein and p-Tau.

## Methods

### Nilotinib and bosutinib treatment

Transgenic mice harboring the A53T mutation of α-Synuclein [36] and age-matched C57BL/6 mice (WT) were treated with intraperotineal (I.P) injection of either 10 mg/kg nilotinib or 5 mg/kg bosutinib or 3 μL dimethylsulfoxide (DMSO) every other day for 6 weeks. All animal experiments were conducted in full compliance with the recommendations of Georgetown University Animal Care and Use Committee (GUAUC). n=20 animals were used for immunohistochemistry, n=15 for longitudinal studies, n=12 were used for brain and blood extraction, n=20 were used for organ extraction and n=40 were used for drug treatment. All graphs and statistical analyses were performed in Graph Pad Prism Software (Graph Pad Prism Software, Inc. CA. USA). All statistics were performed using ANOVA with Newman–Keuls multiple comparison test and data were expressed as Mean ± SD.

### Tissue collection and Milliplex enzyme-linked immunosorbent assay (ELISA)

Animals were deeply anesthetized with a mixture of Xylazine and Ketamine (1:8), and 50–150 μl of whole blood was collected via cardiac puncture, centrifuged at 15000×g to precipitate blood cells and the supernatant was examined by ELISA. To wash out the remaining blood from vessels and reduce contamination, animals were perfused with 10 ml of 1X saline for 4 min and the brain, spleen, heart, gastrocnemius muscle and small intestine were collected and immediately homogenized in 0.5 ml ELISA buffer. We customized a highly sensitive and unbiased Milliplex^®^ MAP Kit (Cat # MPXMCYTO-70K, Millipore) with color-coded microspheres (beads) and fluorescent dyes, which through precise concentrations, the beads can simultaneously and specifically capture mouse cytokines, including IL6, IL-1α, IL-1β, TNF-α, IL-2, IL-3, IL-4, IL-10, VEGF, IFN-γ, CCL2, and CCL5. A total of 25 μL of sample was introduced into a plate containing the microspheres and the reaction mixture was incubated with Streptavidin-PE conjugate and the reporter molecule as described in the manufacturer’s protocol. Using a Luminex^®^ machine, microspheres are first passed through a laser which excites the internal dyes making the microspheres; and a second laser that excites the PE, which is the fluorescent dye on the reporter molecule, and then a high speed digital-signal processor identifies each individual microsphere and quantifies the bioassay.

### Immunohistochemistry of brain sections

Animals were deeply anesthetized with a mixture of Xylazine and Ketamine (1:8), washed with 1X saline for 1 min and then perfused with 4% paraformaldehyde (PFA) for 15–20 min. Brains were quickly dissected out and immediately stored in 4% PFA for 24 h at 4°C, and then transferred to 30% sucrose at 4°C for 48 h. Brains were cut using a cryostat at 4°C into 20-micron-thick coronal sections and stored at -20°C. Immunohistochemistry was performed on 20 μm-thick sections. Astrocytes were probed (1:200) with monoclonal anti-GFAP antibody (Millipore Corporation, USA), and microglia were probed (1:200) with IBA-1 polyclonal antibody (Wako, USA). Dendritic cells and/or microglia were probed (1:200) with CD11b polyclonal antibodies (Thermo Fisher, USA). Nuclear staining with 4′,6-Diamidino-2-Phenylindole (DAPI) was performed according to manufacturer’s protocols (Life Technologies, USA).

### Stereological methods

Stereological methods were applied by a blinded investigator using unbiased stereology analysis (Stereologer, Systems Planning and Analysis, Chester, MD) to determine the total positive cell counts in 20 striatal fields on at least 10 brain sections (~400 positive cells per animal) from each animal. These areas were selected across different regions on either side from the point of injection and all values were averaged to account for the gradient of staining across 1 mm radius from the point of injection. An optical fractionator sampling method was used to estimate the total number of positive cells with multi-level sampling design. Cells were counted within the sampling frame determined optically by the fractionator and cells that fell within the counting frame were counted as the nuclei came into view while focusing through the z-axis.

### Caspase-3 fluorometric activity assay

To measure caspase-3 activity in the animal models, we used EnzChek^®^ caspase-3 assay kit #1 Invitrogen) on cortical extracts and Z-DEVD-AMC substrate and the absorbance was read according to manufacturer’s protocol.

### CX3CL1 ELISA

Mouse CX3CL1 (Cayman) ELISA was performed using 50 μl (1 μg/μl) total brain or blood lysates, which was detected with CX3CL1 primary antibody (3 h) and 100 μl anti-rabbit antibody (30 min) at RT. Extracts were incubated with stabilized Chromogen for 30 min at RT and solution was stopped and read according to manufacturer’s protocol.

### Human α-Synuclein and p-Tau ELISA

Human α-Synuclein and p-Tau ELISA were performed using 50 μl (1 μg/μl) of brain lysates detected with 50 μl primary antibody (3 h) and 100 μl anti-rabbit secondary antibody (30 min) at RT. α-Synuclein levels were measured using human specific ELISA (Invitrogen) according to manufacturers’ protocols. p-Tau was measured using specific p-Tau at serine 396 according to manufacturer’s protocol.

## Results

### Age-dependent alterations of brain immunity in A53T mice

**Figure 1:**
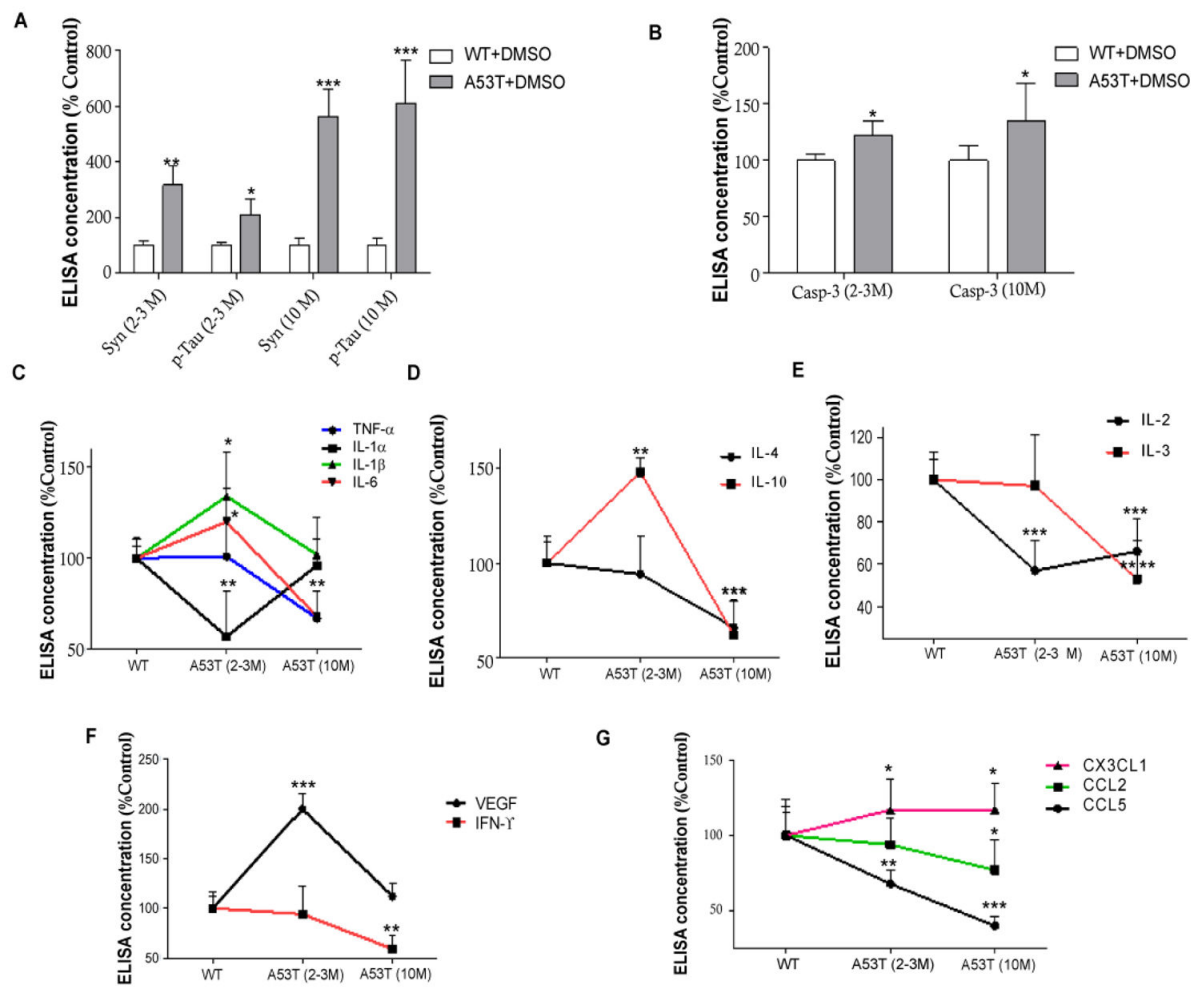
Age-dependent alterations of brain immunity in A53T mice Histograms represent ELISA levels of **A)** α-Synuclein and p-Tau in the brain of A53T mice, and **B)** shows caspase-3 activity in young and older mice. Graphs represent the levels of mouse A53T brain immune markers, including **C)** pro-inflammatory IL-1α, IL-1β, IL-6 and TNF-α, **D)** anti-inflammatory IL-4 and IL-10, **E)** modulators of immune memory IL-2 and IL3, **F)** VEGF and IFN-γ and **G)** chemokines CCL2, CCL5 and CX3CL1. n= 5 for each strain at each time point. ANOVA, Neuman Keuls, Mean±SD, * indicates significantly different than WT with p<0.05, **p<0.01, ***p<0.001, **** p<0.0001.

Age-dependent studies (Figure 1A) showed that A53T mice accumulate a significant level of α-Synuclein (n=5, p<0.01) and serine 396 p-Tau (n=5, p<0.05) as early as 2–3 months of age (Figure 1) compared to wild type (WT) C57BL/6 mice; and these levels increase further at 10 months (n=5, p<0.001), suggesting α-Synuclein and p-Tau accumulation in total brain extracts. It is important to mention that no α-Synuclein or p-Tau is expressed in the substantia nigra in A53T mice [21,36]. Caspase-3 activation was also significantly increased in young (2–3 months) and older (10 months old) A53T mice (Figure 1B, n=5, p<0.05).

### Age-dependent loss of modulators of the immunological memory

We used a highly sensitive unbiased milliplex ELISA to simultaneously measure changes in inflammatory markers in the whole blood and total brain lysates of A53T mice. Significant increases in brain pro-inflammatory cytokines, including interleukin (IL)-6 and IL-1β (n=5, p<0.05), and a decrease in IL-1α (p<0.01) were detected in 2–3 months A53T old mice compared to WT (Figure 1C). No differences were observed in inflammatory profiles between young and older WT mice in these experiments, so WT mice were presented as one age group. No changes in tumor necrosis factor-α (TNF-α) were observed in young A53T (2–3 months) mice, but TNF-α significantly dropped at 10 months (p<0.01). Pro-inflammatory cytokines, including IL-1α and 1β returned to WT level (Figure 1C) and IL-6 significantly decreased below WT levels (n=5, p<0.001). IL-10 in A53T mice was significantly increased (Figure 1D, n=5, p<0.01) at 2–3 months, while both IL-4 and IL-10 were significantly decreased at 10 months (p<0.001), suggesting loss of immunosuppression after prolonged periods of α-Synuclein and p-Tau accumulation. IL-2, which is implicated in T-cell (CD4^+^ and CD8^+^) proliferation, was also significantly decreased at 2–3 months (Figure 1E, n=5, p<0.001) and both IL-2 (p<0.001) and IL-3 (p<0.0001), which may stimulate proliferation of myeloid lineage cells [37], were significantly decreased at 10 months in A53T mice (Figure 1E) compared to WT. Furthermore, interferon (IFN)-γ which is also critical for innate and adaptive immunity [38,39], was significantly decreased at 10 months (Figure 1F, n=5, p<0.01), while Vascular Endothelial Growth Factor (VEGF), which is secreted by endothelial cells and stimulates vasculogenesis and angiogenesis in response to brain injury [40–44] was significantly increased (p<0.001) at 2–3 months in A53T mice and returned to WT level at 10 months (Figure 1F).

Chemokine (C-C motif) ligand 2 (CCL2) or the monocyte chemotactic protein-1 (MCP-1) recruits monocytes, memory T cells, and dendritic cells to the sites of injury [45,46]. CCL5 is another chemotactic for T cells, eosinophils, and basophils, and plays an active role in recruiting leukocytes into inflammatory sites [47,48]. CCL5 was significantly decreased in the brain of 2–3 months old A53T mice (Figure 1G, n=5, p<0.01), and further decrease was observed at 10 months in both CCL2 (n=5, p<0.05) and CCL5 (p<0.001) compared to WT. Soluble fractalkine (CX3CL1) attracts T cells and monocytes, while the cell-bound chemokine promotes adhesion of leukocytes to activated endothelial cells [49]. Importantly, several reports indicated a relationship between CX3CL1, which is expressed on neurons, and microglia activity in neurodegeneration [50,51], particularly in relation to Tau pathology [52,53]. Interestingly, CX3CL1 was significantly increased in the brain of 2–3 months old A53T mice (Figure 1G, n=5, p<0.05) and remained high at 10 months of age compared to WT.

### Differential profiles of systemic and central immunity in A53T mice

**Figure 2:**
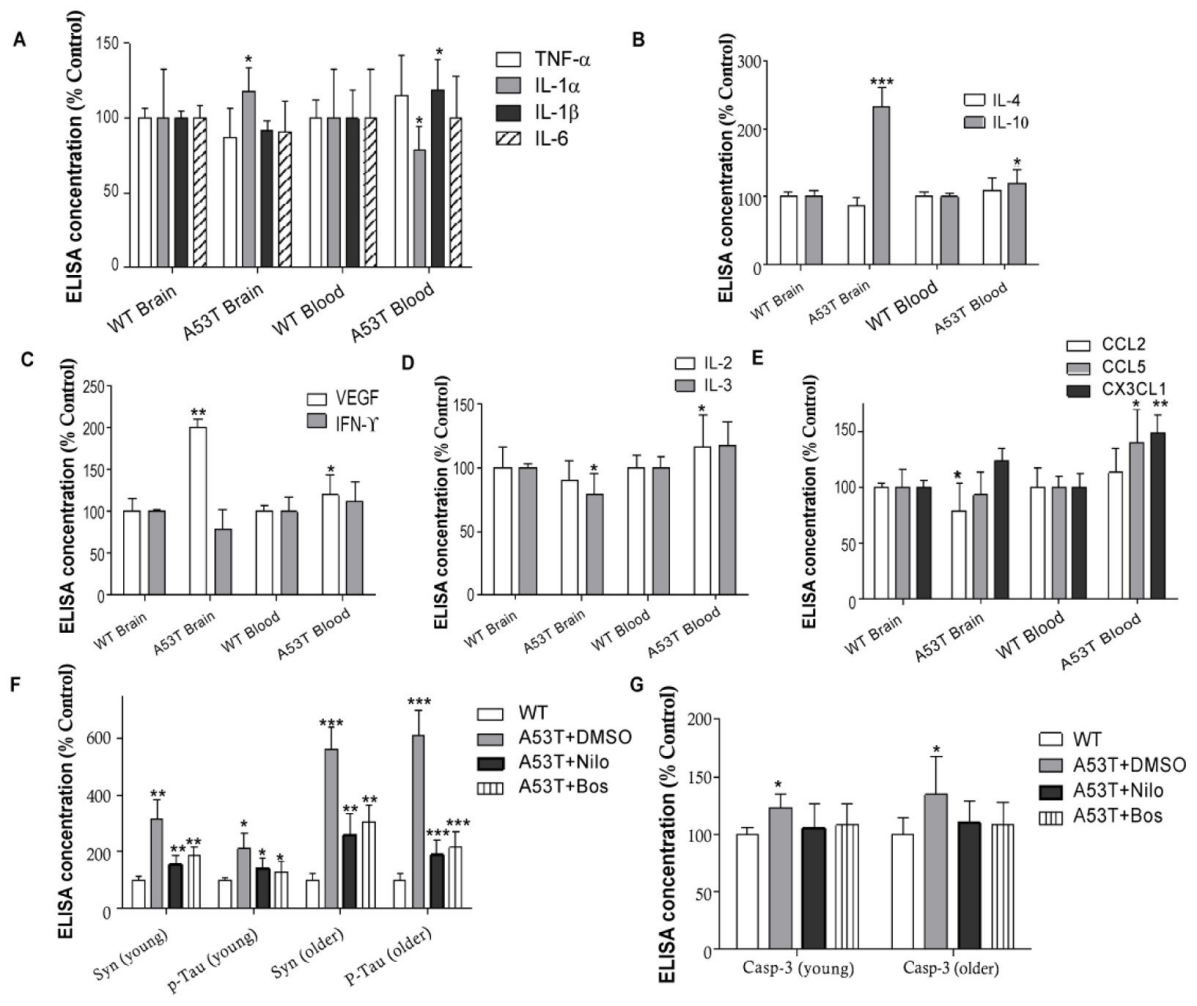
Differential levels of immune markers in A53T brain and blood Histograms represent levels of **A)** pro-inflammatory cytokines IL-1α, IL-1β, IL-6 and TNF-α in the brain and total blood of A53T mice, **B)** anti-inflammatory cytokines IL-4 and IL-10, **C)** modulators of immune memory IL-2 and IL3, **D)** VEGF and IFN-γ and **E)** chemokines CCL2, CCL5 and CX3CL1. n=4 for each strain at each time point. ANOVA, Neuman Keuls, Mean±SD, * indicates significantly different than WT with p<0.05, **p<0.01, ***p<0.001. Histograms represent ELISA levels of F) α-Synuclein and p-Tau and G) caspase-3 activity in the brain of A53T mice treated I.P with 10 mg/kg nilotinib or 5 mg/kg bosutinib or 3 μL DMSO every other day for 6 weeks. n=4 for each strain at each time point. ANOVA, Neuman Keuls, Mean±SD, * indicates significantly different than WT with p<0.05, **p<0.01, ***p<0.001.

Because we observed increased levels of inflammatory cytokines in 2–3 months old A53T mice inflammatory markers were measured in as early as 1–2 month old mice in total blood and brain lysates. Only IL-1α was significantly increased in A53T brains (Figure 2A, n=4, p<0.05) compared to WT, while IL-1α was decreased (p<0.05) and IL-1β was increased in A53T blood (Figure 2A, p<0.05) compared to WT. IL-10 was also significantly increased in the brain (Figure 1B, n=4, p<0.001) and the blood (p<0.05), but IL-4 did not change. A53T brain levels of VEGF were significantly increased compared to WT (Figure 2C, n=4, p<0.01) and IFN-γ was decreased (p<0.05) compared to WT. VEGF levels in total blood of A53T mice were increased (p<0.05) compared to WT. These data suggest changes in the blood immune profile simultaneously with alterations of CNS immune markers. However, while IL-3 was decreased in the A53T brain (Figure 2D, n=4, p<0.05) compared to WT, IL-2 was significantly increased in the blood (p<0.05) compared to WT, suggesting enhanced adaptive immunity in the blood. Additionally, CCL2 was significantly decreased in A53T brains (Figure 2E, n=4, p<0.05) compared to WT, but significant increases in CCL5 (p<0.05) and CX3CL1 (p<0.01) were observed in the A53T blood compared to WT, suggesting altered systemic chemotactic activities.

Nilotinib and bosutinib decrease CNS and peripheral levels of α-Synuclein and p-Tau. Transgenic A53T mice were intraperotineally (I.P) injected with either 10 mg/kg nilotinib or 5 mg/kg bosutinib or 3 μL dimethylsulfoxide (DMSO) every other day for 6 weeks as we previously described [20–22,30]. We previously demonstrated that nilotinib and bosutinib penetrate the brain, inhibit Abl activity and induce autophagic clearance of α-Synuclein and p-Tau in A53T mice and lentiviral gene transfer models [20,21,30,32,33].

**Figure 3:**
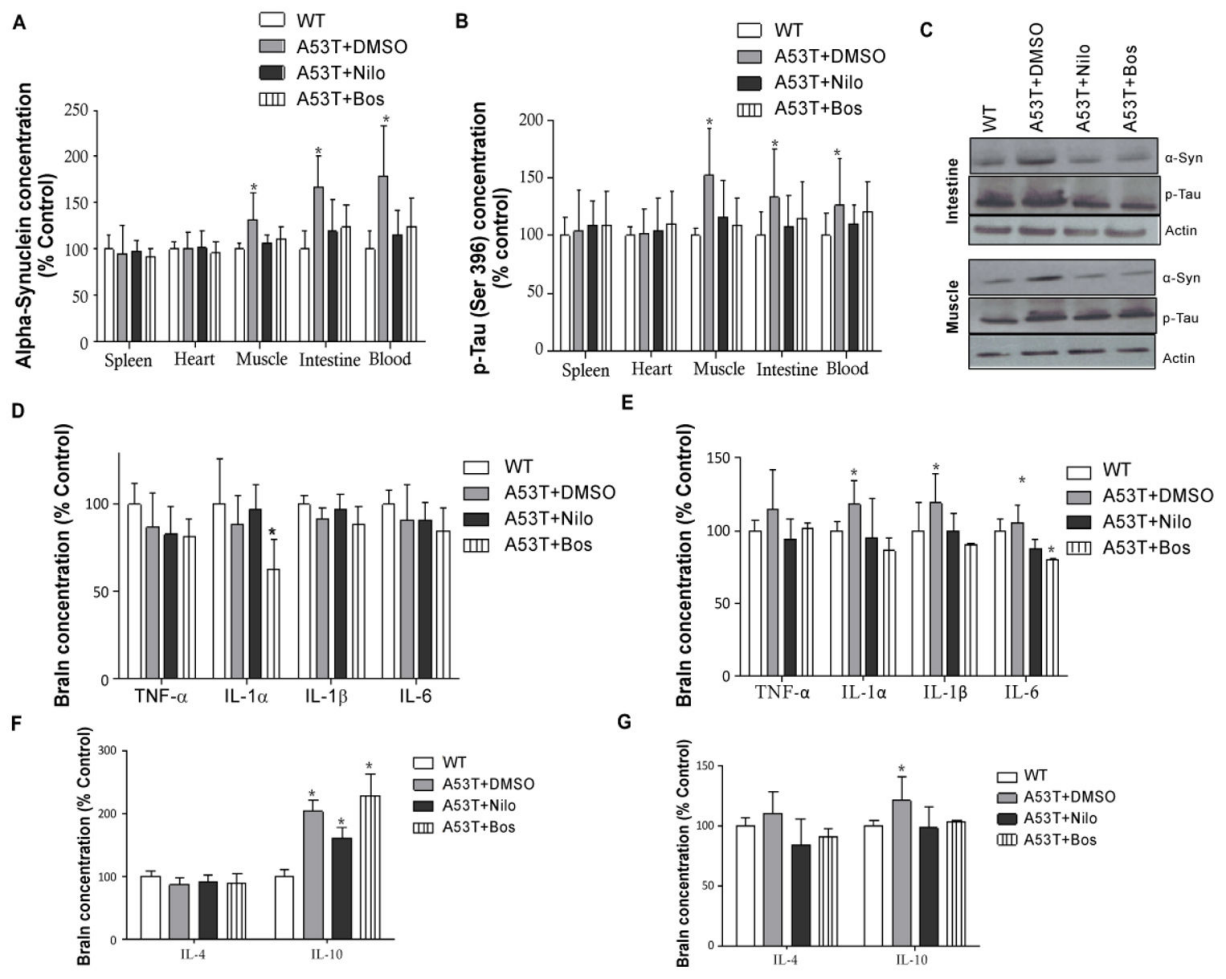
Nilotinib and bosutinib decrease CNS and peripheral levels of α-Synuclein and p-Tau and modulate cytokine levels Histograms represent ELISA levels of **A)** α-Synuclein and **B)** p-Tau in the spleen, heart, muscle, intestine and blood of A53T mice treated I.P with 10 mg/kg nilotinib or 5 mg/kg bosutinib or 3 μL DMSO every other day for 6 weeks. **C)** Western blot analysis on 10% S NuPAGE gel showing analysis of muscle and intestine homogenized in 1xSTEN buffer to compare nilotinib and bosutinib effects with DMSO in A53T mice. **D)** brain and **E)** blood levels of pro-inflammatory IL-1α, IL-1β, IL-6 and TNF-α. **F)** brain and **G)** blood levels of anti-inflammatory IL-4 and IL-10. n= 5 for each strain at each time point. ANOVA, Neuman Keuls, Mean±SD, * indicates significantly different than W, p<0.05.

Here we show the effects of TKIs on α-Synuclein and p-Tau in brain and peripheral tissue in 2–3 months old A53T and WT mice treated with TKIs every other day for 6 weeks. No differences in immunological profiles were observed in WT mice between DMSO, nilotinib and bosutinib treatment, so WT data were presented as only WT. As we previously demonstrated, α-Synuclein was significantly increased (Figure 2F, n=4, p<0.01) in A53T brains compared to WT, and this increase was greater (n=4, p<0.001) in older mice. However, both nilotinib and bosutinib reduced α-Synuclein levels (p<0.01) in young as well as older (p<0.01) A53T mice, which remained higher than WT. p-Tau (ser 396) was also significantly increased (Figure 2F, n=4, p<0.05) in young A53T brains compared to WT, and this increase was greater (n=4, p<0.0001) in older mice. Nilotinib and bosutinib reversed p-Tau levels (p<0.05) in young as well as older (p<0.001) A53T mice, which remained higher than WT. Nilotinib and bosutinib also prevented the increase of caspase-3 activity that was detected in young and old A53T mice treated with DMSO (Figure 2G, p<0.05) compared to WT mice. Both nilotinib and bosutinib significantly reduced p-Tau levels, which remained higher than control. α-Synuclein was also significantly increased in 3–4 month old A53T mice peripheral tissues, including muscle (gastrocnemius), small intestine and blood (Figure 3A, n=4, p<0.05) compared to WT, and nilotinib and bosutinib reversed α-Synuclein levels. Similarly, p-Tau levels were also significantly increased in muscle, small intestine and blood (Figure 3B, n=4, p<0.05) of 3–4 months old A53T mice compared to WT, and nilotinib and bosutinib reduced p-Tau. No effects of TKIs were observed on total α-Synculein or p-Tau levels in WT mice. The decrease in α-Synuclein and p-Tau (threonine 231) levels were further verified by WB analysis that show a significant increase in α-Synuclein and p-Tau in the small intestine and gastrocnemius muscle of A53T mice with DMSO compared to WT (Figure 3C, p<0.05, n=4). However, nilotinib and bosutinib reduced the levels of both α-Synuclein and p-Tau back to WT levels, suggesting TKI effects on peripheral tissue.

**Figure 4:**
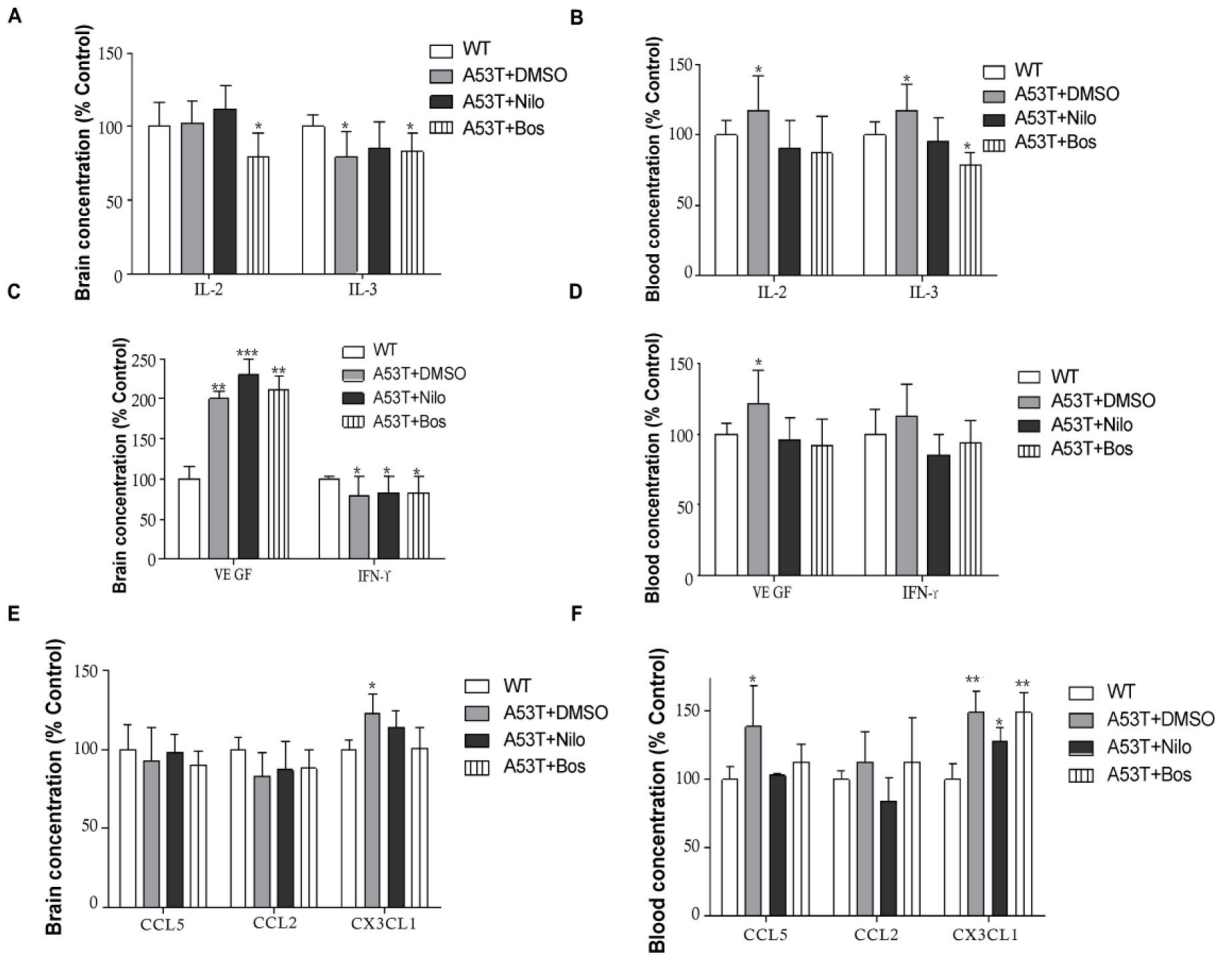
Nilotinib and bosutinib modulate changes in the blood immunological profiles of A53T mice Histograms represent ELISA levels in A53T mice treated I.P with 10 mg/kg nilotinib or 5 mg/kg bosutinib or 3 μL DMSO every other day for 6 weeks in **A)** brain and **B)** blood levels of modulators of immune memory IL-2 and IL3, **C)** brain and **D)** blood VEGF and IFN-γ and **E)** and **F)** blood levels chemokines CCL2, CCL5 and CX3CL1. n=5 for each strain at each time point. ANOVA, Neuman Keuls, Mean±SD, * indicates significantly different than WT with p<0.05, **p<0.01.

Nilotinib and bosutinib modulate changes in blood immunological profiles of A53T mice. To determine the effects of α-Synuclein and p-Tau clearance in 1–2 months old mice, immune markers were measured in whole blood and total brain lysates after 6 week trials with TKIs. Only IL-1α was significantly reduced in the A53T brain (Figure 3D, n=4, p<0.05) compared to WT, and nilotinib and bosutinib reversed this effect in A53T but had no effects on WT mice (data not shown). However, the levels of IL-1α and IL-1β was significantly increased in the A53T blood (Figure 3F, n=5, p<0.05) compared to WT, and nilotinib and bosutinib reversed IL-1α and 1β levels back to WT, and significantly decreased IL-6 (p<0.05) compared to WT. Moreover, IL-10 was significantly increased in the A53T brain (Figure 3F, n=4, p<0.05) compared to WT, but neither nilotinib nor bosutinib altered the level of brain IL-10 in A53T brains, but they reversed it back to WT in the blood (Figure 3G, n=4, p<0.05), suggesting that TKIs may be able to differentially modulate pro-and anti-inflammatory innate immune changes in the blood and the brain. Furthermore, bosutinib significantly reduced IL-2 in the A53T brain (Figure 4A, n=5, p<0.05) compared to DMSO and WT. IL-2 was significantly increased in A53T blood (p<0.05) compared to WT, and both nilotinib and bosutinib reversed the increase of IL-2 in the blood of A53T mice back to WT level (Figure 4B, n=4, p<0.05). Nilotinib and bosutinib did not change the decrease of IL-3 levels in A53T brains (Figure 4A) but nilotinib reversed IL-3 increase in A53T blood (Figure 4B, n=4, p<0.05) and bosutinib reduced it below WT levels (p<0.05). Nilotinib and bosutinib did not affect the alteration of VEGF and IFN-γ in A53T brains compared to WT (Figure 4C, n=4, p<0.05), but they reversed the significant increase of VEGF in A53T blood compared to WT (Figure 4D, n=4, p<0.05). No significant changes were observed in CCL2 and CCL5 in A53T compared to WT brains (Figure 4E, n=4), but CX3CL1 was increased in A53T brains compared to WT (Figure 4E, n=4, p<0.05) and nilotinib and bosutinib reversed this increase back to WT level. However, CCL5 was significantly increased in A53T blood compared to WT (Figure 4F, n=4, p<0.05) and nilotinib and bosutinib abrogated this effect. Blood CX3CL1 was significantly increased in A53T blood compared to WT mice (Figure 4F, n=4, p<0.001) and nilotinib and bosutinib failed to change CX3CL1 levels in the blood.

**Figure 5:**
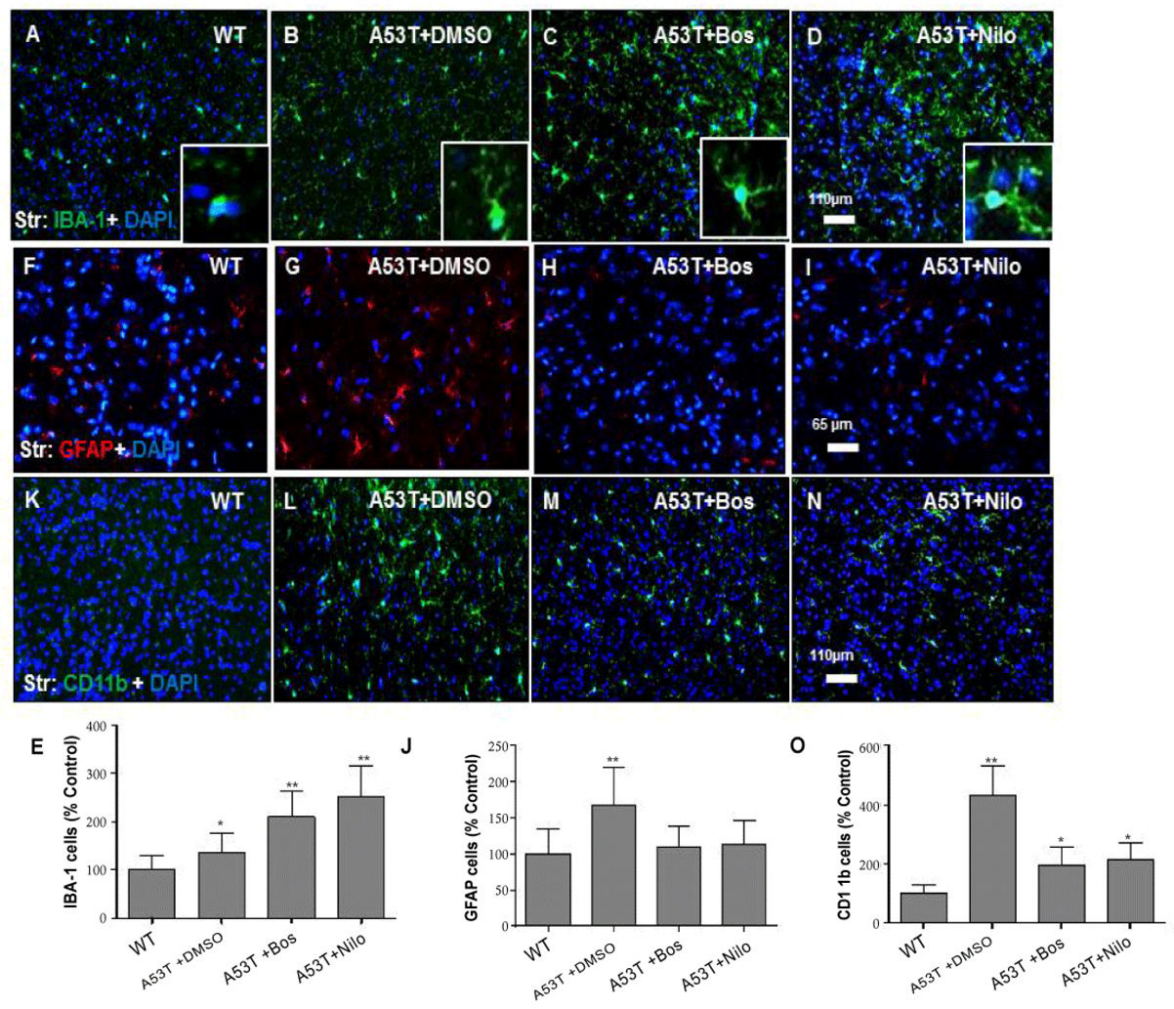
Nilotinib and bosutinib alter microglia morphology and reduce the number of astrocyte and dendritic cells Coronal 20 μm thick brain sections show IBA-1 and nuclear DAPI staining of microglia in the striatum of **A)** WT mice, **B)** A53T mice treated with DMSO, insert is higher magnification, **C)** A53T mice treated with bosutinib, insert is higher magnification, and **D)** A53T mice treated with nilotinib, insert is higher magnification. **E)** histograms represent stereological quantification. Coronal 20 μm thick brain sections show GFAP and nuclear DAPI staining of astrocytes in the striatum of **F)** WT mice, **G)** A53T mice treated with DMSO, **H)** A53T mice treated with bosutinib, and I) A53T mice treated with nilotinib. **J)** histograms represent stereological quantification. Dendritic cells stained with CD11b and nuclear DAPI labeling in the striatum of **K)** WT mice, **L)** A53T mice treated with DMSO, **M)** A53T mice treated with bosutinib, and **N)** A53T mice treated with nilotinib. **O)** histograms represent stereological quantification. n=5 for each strain at each time point. n=4, ANOVA, Neuman Keuls, Mean±SD, * indicates significantly different than WT with p<0.05, **p<0.01.

Nilotinib and bosutinib alter microglia morphology and reduce astrocyte and dendritic cells count. Immuno-histological staining showed increased number of ionized calcium-binding adapter (IBA)-1 positive microglia in the striatum of 3–4 months old A53T mice (Figure 5B, n=4) compared to WT (Figure 5A, n=4). Stereological counting showed a significant increase in microglial number in A53T striatum compared to WT (Figure 5E, n=4, p<0.05), and either bosutinib (Figure 5C, n=4) or nilotinib (Figure 5D, n=4) significantly increased the number of IBA-1-positive microglia (Figure 5E, p<0.01) compared to DMSO. Interestingly, microglia predominantly displayed an amoeboid-like morphology in DMSO treated A53T striatum (Figure 5B, insert) but this morphology appeared ramified after bosutinib (Figure 5C, insert) and nilotinib (Figure 5D, insert) treatment. Glial Fibrillary Acid Protein (GFAP) staining showed increased number of reactive astrocytes in the striatum of 3–4 months old A53T mice (Figure 5G, n=4) compared to WT (Figure 5F, n=4). Stereological counting showed a significant increase in reactive astrocytes in A53T striatum compared to WT (Figure 5J, n=4, p<0.01), and either bosutinib (Figure 5H, n=4) or nilotinib (Figure 5I, n=4) reversed GFAP staining back to WT levels. CD11b staining showed increased number of dendritic cells in the A53T striatum (Figure 5L, n=4) compared to WT (Figure 5K, n=4) and stereological counting showed a significant increase in CD11b-positive cells in A53T compared to WT (Figure 5O, n=4, p<0.001), and bosutinib (Figure 5M, n=4) or nilotinib (Figure 5N, n=4) reversed CD11b staining, which remained higher than WT levels (p<0.05).

## Discussion

These studies provide insights into the systemic immune response and its potential role in modulating innate and adaptive immunity in the brain in α-Synucleinopathies. α-Synuclein and p-Tau may induce a peripheral inflammatory response in parallel with CNS immune alterations, suggesting that Abl activity and pathogenic proteins (p-Tau and α-Synuclein) function within a common loop; involving local communication between microglia and neurons and global crosstalk between body and CNS immunity. The current studies show age-dependent loss of immunosuppression via IL-10 and IL-4 and increase in caspase-3 activity in A53T brains. The early increase in IL-10 is perhaps due to an anti-inflammatory response, which is lost over time and results in decreased levels of the protective anti-inflammatory response at later stages of α-Synuclein accumulation. At least in the blood, IL-4 stimulates T-cell proliferation and induces differentiation of B cells into plasma cells, thus regulating humoral and adaptive immunity, while decreasing the production of TH1 macrophages, IFN-γ, and IL-12 [54–60]. The presence of IL-4 in extravascular tissues also promotes alternative activation of macrophages, which is coupled with secretion of IL-10 and diminution of inflammation [54–60]. In the brain, however, activation of microglia may either be beneficial or detrimental to neuronal survival. It is possible that the observed increase in activated amoeboid-like microglia in young A53T mice is an early event that leads to production of pro-inflammatory cytokines (IL-6 and IL-1β) and caspase-3 activation [61], but modulation of the immune profile, via TKI, may result in ramified resting microglia phenotype [62], indicating a de-activated state. When microglia encounter α-Synuclein or p-Tau they may actively change phenotype into a classical TH1 state, which is induced by TH1 cytokines such as IL-1, IL-6 and TNFα. A second state of activation associated with the TH2 cytokine profile of IL-4, IL-10, is the alternative activation state in which macrophages promote angiogenesis via increased VEGF levels. The ability of nilotinib and bosutinib to efficiently reduce p-Tau and α-Synuclein both centrally and peripherally may regulate microglia activation in A53T mice, leading to beneficial inflammatory response.

The decreased levels of IL-2 and IL-3 in A53T mice suggest loss of the immunological memory, leading to alterations of adaptive immunity. IL-3 is secreted by basophils and activated T cells to support growth and differentiation of multipotent hematopoietic stem cells into myeloid or lymphoid progenitor cells [37]. In addition, IL-3 stimulates proliferation of granulocytes, monocytes, and dendritic cells [37]. Activated T cells can either induce their own, and that of other, proliferation and differentiation of T cells in collaboration with IL-2 [63,64]. IL-2 is normally produced by T cells during an immune response [65,66]. For example, antigen binding to T cell receptors stimulates the secretion of IL-2 and survival of antigen-specific CD4^+^ and CD8^+^ T cells [67–69]. As such, IL-2 and IL-3 are necessary for the development of T cell immunologic memory, which depends upon the expansion of the number and function of antigen-selected T cell clones. Furthermore, IFN-γ activates macrophages and it is critical for innate and adaptive immunity against viral and intracellular bacterial infections and for tumor control; and its aberrant expression is associated with autoimmune diseases [38,39]. Therefore, the importance of IFN-γ in the immune system is its immunomodulatory effects, which seem to be suppressed in the brain and blood of A53T mice. Furthermore, IFN-γ is produced predominantly by natural killer cells as part of the innate immune response, and by CD4 Th1 and CD8 cytotoxic T cells once antigen-specific immunity develops [38,39]. However, despite the increased level of α-Synuclein and p-Tau in the blood and other tissues, including muscle and small intestine, no endogenous antibodies seem to be produced via T cell activation to protect against the pathogenic increase in amyloid protein in the blood or the brain. This suggests that T cells are either assured that the pathogenic species are not ‘foreign invaders’ or antigenic, or the immune system is incapable of mounting a protective strategy to degrade or eliminate α-Synuclein and p-Tau. However, the non-immunological autophagic clearance of amyloids with nilotinib and bosutinib may restrain the inflammatory response in the absence of immunological memory.

Chemokine brain levels, including CCL2 and CCL5 was decreased, but CX3CL1 concentration remained high independent of the effects of TKI, suggesting altered chemotactic activity in the brain and blood of A53T mice. CCL2 is involved in the neuroinflammatory processes that take place in neurodegeneration [70]. CCL5 recruits T cells, eosinophils, basophils, and leukocytes into inflammatory sites, and induces the proliferation and activation of certain natural-killer cells with the help of particular T-cell-released IL-2 and IFN-γ [47,48]. Therefore, the simultaneous decrease in CCL2, CCL5 as well as IL-2 and IFN-γ, suggests a vicious cycle that reflects failure of immunological counteraction of α-Synuclein and p-Tau in A53T mice. CCL2 and CCL5 play a crucial role in the brain, but their alterations have not been well studied in systemic immunity in neurodegenerative diseases. CCL2 is expressed by neurons throughout the brain [71] and its expression level in glial cells is increased in epilepsy [72,73], brain ischemia [74], AD [75], experimental autoimmune encephalomyelitis (EAE) [76], and traumatic brain injury (TBI) [77]. CCL5 is a Human Immunodeficiency Virus (HIV)-suppressive factor released from CD8^+^ cells [78] and it is implicated in several human diseases [79–82].

It is important to note that the constantly elevated level of CX3CL1 was only decreased in the brain, but not in the blood, in A53T mice treated with nilotinib and bosutinib, suggesting that autophagic clearance of α-Synuclein and p-Tau may lead to attenuation of brain CX3CL1 level. The relationship between CX3CL1 and microglia activity has been extensively studied in several models of neurodegeneration, but fluctuations of soluble blood CX3CL1 have not been demonstrated in previous studies. We previously demonstrated that CX3CL1 levels are differentially altered in Tau gene transfer animal models that also over-express murine phosphorylated α-Synuclein [11,83]. Neurons secrete CX3CL1 [84], which exists in both membrane-bound and soluble forms [85].

The membrane-bound CX3CL1 can serve as an adhesion molecule for leukocytes expressing the fractalkine receptor (CX3CR1) [86] and soluble CX3CL1 can function as both a pro-inflammatory chemo-attractant that activates receptive inflammatory cells [49,87] and an anti-inflammatory [88], neuro-protective agent that reduces neuronal apoptosis [89]. Several findings suggest that deletion of CX3CR1 increases microglia activity in models of acute and chronic neuronal injury [90–93]. Exogenous CX3CL1 is neuro-protective in some models of neuro-inflammation [94,95], and disruption of CX3CL1 signaling causes neurotoxicity in models of systemic inflammation, PD, and amyotrophic lateral sclerosis [96] but protects against neuronal loss in a mouse model of focal cerebral ischemia [97]. Although the relationship between soluble CX3CL1 in peripheral blood and inflammatory diseases of the CNS has not been studied, serum CX3CL1 is increased in patients with multiple sclerosis [89,98], TBI [99] and HIV with CNS complications [100].

In conclusion, these studies demonstrate that nilotinib and bosutinib can reduce the levels of α-Synuclein and p-Tau in peripheral tissues and inside the CNS, and are therapeutic candidates to treat gastrointestinal complications in α-Synucleinopathies and other neurodegenerative diseases. Decreased inflammation may also contribute to less protein accumulation. TKIs may also be used to modulate the peripheral immune profile, which may affect CNS immunity, providing a double-edged strategy to facilitate protein degradation and orchestrate the systemic and CNS inflammatory response, thus mediating beneficial regulation of innate and adaptive immunity. The differential effects of nilotinib and bosutinib on the immune profile may be attributed to their potency to inhibit Abl and/or Src and other tyrosine kinases [34,35]. Finally, Abl and other TKIs should be explored as anti-inflammatory agents that provide combined effects, including amyloid clearance and anti-inflammation in neurodegenerative diseases.
